# Effects of Laser Therapy on Periodontal Status in Adult Patients Undergoing Orthodontic Treatment

**DOI:** 10.3390/diagnostics12112672

**Published:** 2022-11-03

**Authors:** Luminița Lazăr, Timea Dako, Maria-Alexandra Mârțu, Cristina-Ioana Bica, Anamaria Bud, Mircea Suciu, Mariana Păcurar, Ana-Petra Lazăr

**Affiliations:** 1 Department of Periodontology, George Emil Palade University of Medicine, Pharmacy, Science, and Technology of Târgu Mures, 38 Ghe. Marinescu Street, 540139 Târgu Mures, Romania; 2 Department of Odontology and Oral Pathology, George Emil Palade University of Medicine, Pharmacy, Science, and Technology of Târgu Mures, 38 Ghe. Marinescu Street, 540139 Târgu Mures, Romania; 3 Department of Periodontology, Grigore T. Popa University of Medicine and Pharmacy Iasi, Universitatii Street 16, 700115 Iasi, Romania; 4 Department of Pedodontics, George Emil Palade University of Medicine, Pharmacy, Science, and Technology of Târgu Mures, 38 Ghe. Marinescu Street, 540139 Târgu Mures, Romania; 5 Department of Oral Rehabilitation and Occlusology, George Emil Palade University of Medicine, Pharmacy, Science, and Technology of Târgu Mures, 38 Ghe. Marinescu Street, 540139 Târgu Mures, Romania; 6 Department of Orthodontics, George Emil Palade University of Medicine, Pharmacy, Science, and Technology of Târgu Mures, 38 Ghe. Marinescu Street, 540139 Târgu Mures, Romania; 7 Institution Organizing University Doctoral Studies (I. O. S. U. D.), George Emil Palade University of Medicine, Pharmacy, Science, and Technology of Târgu Mures, 38 Ghe. Marinescu Street, 540139 Târgu Mures, Romania

**Keywords:** periodontal status, laser, orthodontic treatment, periodontal pathogens, oral hygiene, gingivitis

## Abstract

Orthodontic treatment with fixed devices should only be indicated in case of a stable, non-active periodontal disease status. Throughout orthodontic treatment, a careful assessment of the periodontal status is advised. Due to its anti-inflammatory and antimicrobial effects, laser therapy is frequently used as an adjunct to classic periodontal therapy. The aim of this study was to evaluate the advantages and limitations of the use of laser therapy on periodontal status during orthodontic treatment. Throughout the 9 months during which this placebo-controlled, single-blind clinical trial was conducted, 32 patients were included in the study, divided into two groups: microscope “+” (patients who observed the bacteria within the dental plaque-sample examination on the screen of a dark-field microscope in real time) and microscope “−” (patients who did not see the oral pathogens using a dark-field microscope). For all patients, using the split-mouth study design, laser therapy was applied to one hemiarch (HL), whereas the other hemiarch received treatment without active light (HC). After one month, by analyzing the main indicators of periodontal health status, we found that the plaque index (PI) and bleeding on probing (BOP) values were significantly decreased after receiving treatment (for PI: HL-p = 0.0005, HC-p = 0.0297; for BOP: HL-p = 0.0121, HC-p = 0.0236), whereas the probing-depth (PD) values remained almost the same as before treatment (HL-p = 1.5143; HC-p = 1.4762). Conclusions: The use of the dark-field microscope proved to be beneficial in sensitizing patients to the presence of bacteria in the oral cavity and motivated them to strictly follow the rules of oral hygiene. Laser treatment can be a valuable aid in periodontal therapy, but only in adjunction with mechanical therapy.

## 1. Introduction

Orthodontic therapy with fixed appliances should only be indicated in conditions of well-maintained clinical periodontal health in intact periodontium or a stable periodontal disease in reduced periodontium. However, during orthodontic treatment, pathological changes may occur in the periodontal tissue, which are initially manifested by gingival inflammation. Untreated, over time, these can lead to the damage of the deep periodontal structures. That is why, throughout orthodontic treatment, a careful assessment of the periodontal status is required by recording the main indicators of periodontal status: plaque index (PI), probing depth (PD), bleeding on probing (BOP), gingival recession (GR), and clinical attachment loss (CAL).

Fixed orthodontic treatment is still the best and most common choice for treating different types of malocclusions [1,2,3,4]. Although the effectiveness of traditional brackets has been recognized worldwide, this therapeutic method still has some disadvantages. Wearing a fixed orthodontic appliance will make it more difficult to maintain oral hygiene and thus favor the accumulation of dental plaque [5,6]. In addition, Yáñez-Vico et al. found that even regular activations can be uncomfortable and inconvenient, which can seriously hinder proper oral hygiene measures [7]. Numerous sites of plaque retention can appear, which leads to the development of white spots of demineralization, caries, or periodontal disease [8,9,10,11].

Patients should carefully brush each bracket and around the wires to remove all traces of plaque in order to reduce the risk of enamel demineralization during treatment [12,13]. Some previous studies have found that treatment with fixed orthodontic appliances will stimulate the growth of subgingival plaque [14,15,16]. Orthodontic appliances affect the microbial composition of the subgingival plaque even during the early stages of orthodontic treatment, increasing the prevalence of periodontal pathogens (*Tanerella forsythia*, *Treponema denticola*, *Fusobacterium nodatum*, *Campylobacter rectus*, *Eubacterium nodatum*, *Eikenella corrodens*, *Capnocytophaga* spp.) but also raising the level of certain molecules responsible for periodontal tissue breakdown such as MMP-8 and MMP-9 [17,18]. The identification of periodontal pathogens (*A. actinomycetemcomitans*, *P. gingivalis*, *P. intermedia*, *T. forsythia*, *T. denticola*) by the polymerase chain-reaction (PCR) technique as well as modified periodontal clinical indexes requires the institution of local mechanical therapy (scaling and root planing (SRP)), associated or not with an antimicrobial treatment [19].

Laser therapy is increasingly used in dentistry due to the advantages it offers. Among them are its anti-inflammatory and antimicrobial action; therefore, nowadays it is frequently employed as an adjunct to non-surgical periodontal therapy [20,21,22,23,24].

There is a number of studies that have questioned the efficacy and clinical advantages of laser therapy, especially when used as an adjunctive procedure [25,26,27]. They claim that the clinical advantages are not significant in terms of improvement of periodontal clinical parameters in the long term, and thus the added cost of this therapy does not justify its use [28,29,30,31].

Most patients undergoing fixed orthodontic treatment have inflammatory changes in the periodontal structures, most frequently determined by the accumulation of bacterial plaque. The general aim of this study was to evaluate the advantages and limitations of the use of laser therapy. The hypothesis of this study was that laser therapy can be used as an adjuvant in improving the periodontal status during orthodontic treatment. The first objective was to confirm this hypothesis by recording the periodontal clinical parameters in patients with fixed orthodontic appliances.

As a secondary objective, we aimed to evaluate whether the dark-field microscope is effective in identifying patients to improve their oral hygiene.

## 2. Materials and Methods

### 2.1. Study Design

The study was conducted as a placebo-controlled, single-blind clinical trial between November 2021 and July 2022.

### 2.2. Selection of Patients

Among the adult patients with fixed orthodontic treatment who presented themselves to CMI Dr. Lazăr Luminița, we selected 32 patients who met the inclusion criteria:

- Age between 20 and 50 years;
- Presence of dento-alveolar disharmony (DAD) with mild crowding (3–7 mm);
- Signs of gingival inflammation and dental plaque accumulation during the orthodontic treatment.

The exclusion criteria were the following:

- The presence of systemic diseases with an impact on the periodontal tissues (diabetes, immunological diseases, acute articular rheumatism, tuberculosis, etc.);
- History of smoking;
- Pregnancy or breastfeeding;
- Antibiotic treatment in the last 6 months;
- The use of anti-inflammatory drugs (NSAIDs) or other medication that might interfere with periodontal status in the last 6 months.

The patients were informed about the working procedure and about the fact that they could leave our study at any time, and signed an informed consent.

### 2.3. Orthodontic Protocol

Patients presenting dentoalveolar disharmony with crowding were examined by a specialist and received orthodontic treatment using standard edgewise brackets with the same slot size for all subjects (0.22) (American Orthodontics, Sheboygan, WI, USA). The following sequence of archwires was used: NiTi size 0.12 when applying the orthodontic device and sizes 0.16 for the first activation, 0.16 × 0.16 for the second, and 0.16 × 0.22 for the third (BioForce, Dentsply Sirona, Charlotte, NC, USA).

### 2.4. Periodontal Protocol

Before starting the orthodontic treatment, an assessment of the periodontal status was conducted by a periodontal specialist, and the application of the device was only carried out under the conditions of a healthy periodontal status.

Patients who, at the activation sessions, showed plaque accumulation and signs of gingival inflammation (gingivitis) were subjected to a new periodontal examination with the recording of the following indices in the patient’s record:

- Plaque index (PI): the presence (+) or absence (−) of bacterial plaque on the buccal, oral, mesial, and distal surfaces, following the application of a plaque-revealing solution. The PI value was calculated by dividing the sum of all surfaces with dental plaque by the total number of surfaces examined, multiplied by 100.
- Bleeding on probing (BOP): determined by the presence (+) or absence (−) of bleeding when probing the gingival sulcus. The BOP value was calculated by reporting the number of sites that showed bleeding on probing to the number of sites examined, multiplied by 100.
- Probing depth (PD): the distance from the gingival margin to the apical limit of the gingival sulcus, measured in 6 places (mesio-buccal/centro-buccal/disto-buccal/mesio-oral/centro-oral/disto-oral) with a constant palpation force.

One of the inclusion criteria for our study was for patients to present dental-plaque accumulation (PI > 25%) and signs of gingival inflammation (BOP > 10%) at the activation sessions of the orthodontic appliance. During the clinical evaluation, patients who had values of PD > 3 mm were excluded from our study and referred to a complex periodontal consultation and treatment.

According to the values of these indices and the age and gender of the patients, they were divided into two groups: Group 1 (G1)—microscope “−” and Group 2 (G2)—microscope “+” so that the groups were as homogeneous as possible. Subgingival plaque samples were collected from each hemiarch (HC—control hemiarch and HL—laser-treated hemiarch) with a Gracey curette from the areas with the most significant buildups and analyzed under a dark-field microscope. Plaque samples were examined for the G2 group in the presence of the patient, who observed the results on the screen attached to the microscope. Patients in the G1 group did not have access to this information.

With the help of the dark-field microscope, we highlighted, based on their morphological characters, the bacteria that colonize the gingival groove. The quantitative analysis was subjective, but as it was carried out by three team members, the results could be realistic.

Patients were instructed again on oral-hygiene methods and then supra- and subgingival scaling and professional brushing was conducted, followed by the laser protocol.

### 2.5. Laser Protocol

**Figure 1:**
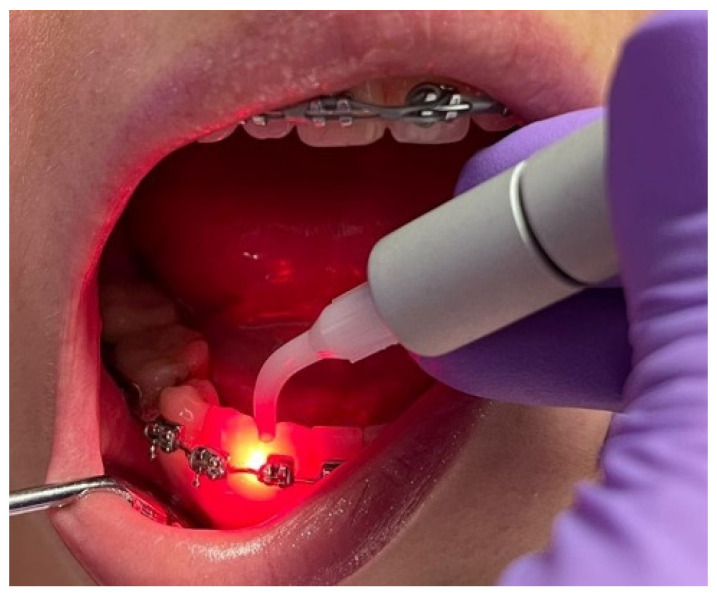
Laser therapy applied to a patient with a fixed orthodontic device.

Another team member performed randomized laser therapy on the right or left hemiarch (HL) for each patient, chosen randomly. Laser therapy was applied on a hemiarch from the incisor to the molar (HL). On the other side, on the control hemiarch (HC), the same protocol was conducted but without active light. Patients were not informed that only one hemiarch benefited from laser therapy. During the therapy, both the patient and the research-team member wore protective glasses (Figure 1).

We opted for the arrangement of the control group and the laser group by dividing the dental arches into two (HC, HL) for each patient and not by dividing the patients into two groups so that the oral-hygiene habits of the patients did not influence our results.

Laser therapy was performed with a diode dental laser (Litemedics Prime, New Delhi, India) with a power of 1 Watt in a pulsed system and operating wave of 980 nm using the Periodontology work mode. The 320-micrometer optical fiber was inserted into the gingival sulcus and moved in a mesio-distal direction, on both the buccal and oral surfaces, for 20 s.

### 2.6. Evaluation of Laser Therapy

One month after the laser therapy, before the activation of the orthodontic appliance, a re-evaluation of the periodontal status (PI, BOP, PD) and the harvesting of dental plaque from each hemiarch (HL, HC) was performed. The values were compared with those previously recorded.

### 2.7. Statistical Analysis

All data were collected in Microsoft Excel worksheets (Microsoft Corporation, 2018). The statistical analysis was carried out in GraphPad Prism version 8.0.0 for Windows (GraphPad Software, San Diego, CA, USA). For each group of data, descriptive statistics such as mean, standard deviation, median, minimum, and maximum value were assessed. Data normality was determined using the Kolmogorov–Smirnov test. The difference regarding indicators of periodontal status, including plaque index (PI), probing depth (PD), and bleeding on probing (BOP) in the G1 and G2 groups for HC and HL, were determined using the Chi-square and Fischer tests. The chosen significance level was set at 0.05.

## 3. Results

Patients included in this study based on the inclusion and exclusion criteria were aged between 20 and 46 years. Group 1 included nine women with an average age of 32 and seven men with an average age of 29; Group 2 included eight women with an average age of 32 and eight men with an average age of 30.

The mean values recorded for PI, BOP, and PD for each group are presented in Table 1.

**Figure 2:**
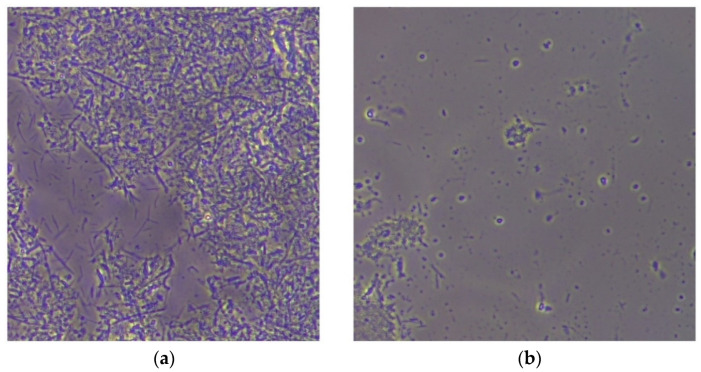
Dark-field microscope images of oral bacterial flora before treatment (**a**) and after treatment (**b**).

**Figure 3:**
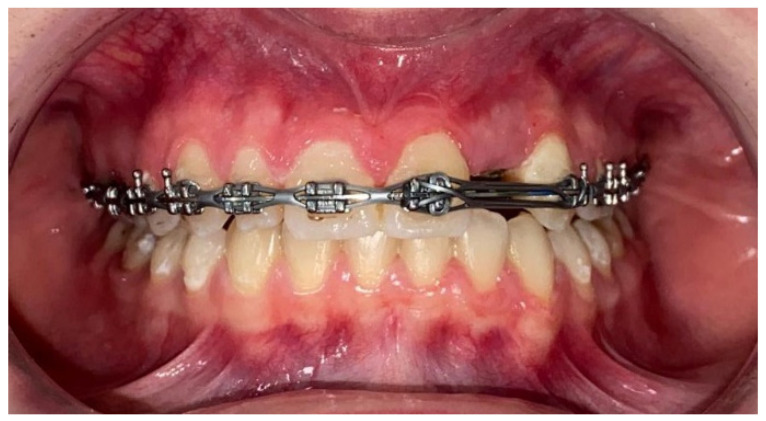
Patient wearing fixed orthodontic appliances. The accumulation of dental plaque can be observed along the gingival line and around the orthodontic appliances.

Despite all these shortcomings, this investigation allowed us to observe in all patients at time T0 a rich bacterial load, represented especially by bacillary forms. At time T1, we observed a quantitative reduction in the bacterial flora, greater for G2 than for G1, and the predominance of cocci (Figure 2 and Figure 3).

At time T0 for all patients, we recorded high PI values, with an average of 72.76 in G1 and 71.42 in G2. After a rigorous professional dental cleaning and educating the patient on anti-plaque measures, PI values recorded at T1 were lower for all patients. The index values between HC and HL within G1 and G2 were not statistically different in either T0 (*p* = 1.000) or T1 (*p* = 0.8388). Comparing the values obtained for PI in G2 patients, who saw the analysis of the bacterial plaque under the dark-field microscope at time T1, they were significantly lower in both HC (*p* = 0.0297) and in HL (0.0005) versus those of G1 patients.

According to the bleeding-on-probing index (BOP), we concluded that all the patients included in our study presented generalized gingivitis at time T0, with values higher than 30% (G1—67.85%, G2—66.96%). The mean value of BOP at T1 in G1 for HC was 21.42%, whereas for HL it was 11.60%. In G2, the mean BOP values were 7.14% at HC and 1.78% for HL. The comparison between BOP index values of T0 and T1 for G1 and G2 revealed statistically significant differences for both HC (*p* = 0.0236) and HL (*p* = 0.0121). No statistically significant difference was found when comparing the values of the BOP index in the control and laser hemiarches for G1 and G2 for either time T0 (*p* = 1.000) or time T1 (*p* = 0.8149).

The mean probing depth (PD) in G1 was 2.87 mm at time T0 compared to T1, which was 2.31 mm HC and 2.18 mm for HL. For G2, the mean values of PD at T0 were 2.81 mm, and at T1, 2.12 mm (HC) and 2.06 mm (HL). The difference between the PD index values for T0, T1, and G1 and G2 were not statistically significant for either HC (*p* = 1.4762) or HL (*p* = 1.5143). When comparing the PD index values between HC and HL for both groups, there was no statistically significant difference between T0 (*p* = 1.4329) and T1 (*p* = 1.5143). 

## 4. Discussion

In patients wearing fixed orthodontic appliances, we frequently observed the accumulation of dental plaque. This is due to either an incorrect brushing technique, or to the fact that brushing is made more difficult by the presence of the orthodontic appliance. The persistence of supra- and subgingival dental-plaque buildup causes the onset of inflammatory changes in the periodontal structures (gingivitis), which, if left untreated for a longer period, evolve into periodontitis.

More and more authors regard laser therapy as a valuable therapeutic alternative to reduce bacterial flora.

The purpose of a study conducted at the University of California in 2017 was to evaluate and demonstrate the potential effectiveness of laser therapy on biofilm-infected tissue. Staphylococcus epidermidis was allowed to proliferate on pig skin ex vivo until mature biofilm was formed and then subjected to laser therapy. Using the swab technique and colony counting, the bacterial load between control and treated samples was compared. Laser therapy reduced the bacterial load by 69% (*p* = 0.008). Imaging showed that biofilm coverage was reduced by 52% and mean cluster size was significantly reduced (*p* < 0.001). The authors concluded that laser therapy is a new treatment for infected wounds that allows rapid biofilm destruction by removing bacteria and increasing the susceptibility of the remaining biofilm to topical antibiotics [32].

Rupel et al. evaluated the influence of laser irradiation on culture medium with Pseudomonas aeruginosa. Irradiation of the mature biofilm with a high blue laser significantly reduced the biomass due to cell detachment from the surface. By increasing the irradiation intensity and applying it to the top and bottom of the biofilm, the entire biomass was detached from the surface almost completely and was washed away with no detectable bacteria living. Using an infrared laser source, biomass reduction was detected, but not as drastic as the one observed in samples exposed to the blue laser. The obtained results indicate oxidative stress as a relevant mechanism by which blue laser light exercises its antimicrobial effect [33].

The development of laser technology and the discovery of its antimicrobial effects have made laser therapy a new method used in periodontal treatment. Gojkov-Vukelic et al estimated the efficiency of diode-laser application in reducing periodontal pockets. To do so, they collected a sample of subgingival plaque for biomolecular analysis (real-time PCR method) before laser irradiation of periodontal pockets immediately after irradiation and 3 months after irradiation. The results showed that there was a statistically significant decrease in the bacteria tested (*Aggregatibacter actinomycetemcomitans* and *Porphyromonas gingivalis*) immediately after treatment and at the 3-month examination compared to the values for the same bacteria before treatment. Based on the obtained results, the authors concluded that diode-laser irradiation reduces the number of periodontal pathogens and that laser therapy can be considered a useful and effective additional method in the treatment of periodontal disease [34].

To assess the extent to which laser therapy could help reduce the total number of bacteria, Grzech-Leśniak et al. carried out microbiological analyses and clinical assessments at baseline, 3 months, 3 months and 1 week, and 6 months. Patients were divided into group G1, which received laser therapy with 40 mJ of energy, a frequency of 40 Hz, and a fluence of 63.66 J/cm^2^; and group G2, which underwent mechanical treatment (SRP) and PDT photobiostimulation (635 nm diode laser, 12 J of energy and 30 s irradiation time, a toluidine blue as a photosensitizer (PS), application time of 60 s); and group G3, in which only SRP was performed. A reduction in the percentage of bacteria was found after 3 months for *Prevotella intermedia* (G1 and G2), *Capnocytophaga gingivalis*, and *Eubacterium nucleatum* (G3). A reduction was found after 3 months and 1 week for *Eubacterium nucleatum*, *Treponema denticola*, and *Tannerella forsythia* (G1, G2, and G3); *Prevotella intermedia* (G1 and G2); *Aggregatibacter actinomycetemcomitans*, *Peptostreptococcus micros*, and *Capnocytophaga gingivalis* (G3); and *Prevotella intermedia* (G1 and G2), *Tannerella forsythia* (G2), *Porphyromonas gingivalis*, and *Eubacterium nucleatum* (G2 and G3). A reduction was found for *Peptostreptococcus micros* (G3) after 6 months (*p* < 0.05). The authors concluded that lasers offer an alternative therapy or a complementary non-invasive method in periodontal treatment. The use of laser therapy could help decrease the number of periodontal pathogenic bacteria and keep their number at a stable, low level [35].

Wang et al. compared the detection rate of periodontal pathogens between two types of treatments: Er:YAG laser therapy and conventional mechanical therapy (SRP—scaling and root planing). The results showed a similar improvement for both treatments, except for a lower detection rate of *Porphyromonas gingivalis* in the SRP group, 6 months after treatment. Therefore, the authors believe that for medium periodontal pockets, SRP may still be the preferred choice, whereas for deep periodontal pockets, Er:YAG laser treatment could be an effective alternative [36]. Other studies have suggested that Er:YAG laser treatment has bactericidal efficacy comparable to SRP [37,38].

The aim of a study conducted by Calderín et al. was to evaluate the clinical, anti-inflammatory, and osteoimmunological benefits of single (PT) and repeated laser phototherapy (rPT) as adjuvant treatment of inflamed periodontal tissue. PT used in a single or repeated dose does not produce a significant reduction in the tested clinical parameters (*p* > 0.05). However, IL-1β levels in GCF were significantly reduced in the SRP + PT and SRP + rPT groups compared to the SRP group (*p* < 0.05) [39].

Dalvi et al., following a systematic review and meta-analysis, concluded that the efficacy of photodynamic therapy in improving treatment outcomes, when used in the non-surgical management of periodontitis, remains questionable. However, the results from most of the included studies demonstrated the efficacy of photodynamic therapy and that its role is more pronounced when combined with mechanical therapy (SRP) than when alone [40].

Recording periodontal parameters: Plaque index (PI), bleeding on probing (BOP), probing depth (PD), and clinical attachment loss (CAL) are useful in assessing the effectiveness of therapeutic means aimed at improving periodontal status. Sufaru et al. observed at 6 months that both SRP alone and SRP associated with 810 nm diode-laser irradiation and with 5 mg/mL indocyanine green (aPDT) resulted in a significant reduction in all clinical parameters investigated. The combination of SRP with photodynamic therapy produced more significant reductions for BOP, PD, and CAL (*p* < 0.001) than SRP alone, but not for PI. Therefore, aPDT could represent an adjuvant periodontal treatment option for SRP [41].

Recording periodontal parameters has been proven to be useful in appreciating the effectiveness of laser therapy as an adjuvant to non-surgical periodontal therapy in patients with orthodontic treatment [42,43].

In our study, we used the recording of periodontal indices to assess the effectiveness of laser therapy and the use of a microscope with a dark background. In the assessment of the subgingival bacterial flora, the greater reduction for G2 than for G1 is due to the fact that the patients in G2 participated in the dark-field microscope examination. These patients saw in real time, on the microscope screen, the bacteria that were present in their oral cavity and were surprised by their presence. As a result, they were much more compliant with oral-hygiene measures and followed them strictly.

Even though the analysis of the bacterial load using the microscope with a dark background was not quantitative, the study has meaningful practical applicability. By increasing patient compliance regarding oral-hygiene measures, the risk of periodontal complications during orthodontic treatment is reduced.

An element of limitation in our study derives from the fact that there was a small number of patients included in the study and they were of both genders and heterogeneous age. It has been demonstrated that there are significant differences between patients regarding the maintenance of oral hygiene. On the other hand, a mixed group represents a positive point because it allows the results to be generalized. Another limitation is related to the fact that the microscopic examination of the subgingival plaque samples only allowed us to classify the bacteria in the genus based on the morphological characters and not in the species. The quantitative analysis was subjective, but it was performed by three different specialists so as to increase the accuracy of detection.

During orthodontic treatment, the adult patients’ initially healthy periodontal status may undergo pathological changes due to the accumulation of dental plaque. Training the patient to combat the accumulation of dental plaque and the permanent monitoring of the periodontal status throughout the orthodontic treatment are mandatory to prevent the establishment of irreversible forms of periodontal disease.

## 5. Conclusions

1. The use of the dark-field microscope proved to be beneficial in sensitizing patients to the presence of bacteria in the oral cavity and motivating them to strictly follow the rules of oral hygiene.

2. In our study, laser therapy was successfully used as an adjunct to mechanical therapy in order to reduce the bacterial load in the periodontal structures. Its application in patients with orthodontic treatment led to a significant reduction of any signs of gingival inflammation, comparing the laser hemiarch with the control hemiarch. 

3. However, laser therapy requires a better understanding of its mechanism of action by conducting more high-quality studies with larger sample sizes and longer follow-up periods.

## Figures and Tables

**Table 1:** The mean values of PI, BOP, and PD.

Patients	PI (T0)	PI (T1)	BOP (T0)	BOP (T1)	PD (T0)	PD (T1)
G1 HC	72.76	24.99	67.85	21.42	2.87	2.31
G1 HL	72.76	16.35	67.85	11.60	2.87	2.18
G2 HC	71.42	10.26	66.96	7.14	2.81	2.12
G2 HL	71.42	4.90	66.96	1.78	2.81	2.06

PI = plaque index; BOP = bleeding on probing; PD = probing depth; T0 = before treatment; T1 = after treatment; G1 HC = Group 1 control hemiarch; G1 HL = Group 1 laser hemiarch; G2 HC = Group 2 control hemiarch; G2 HL = Group 2 laser hemiarch.

## Data Availability

The data presented in this study are available on request from the corresponding authors. The data are not publicly available due to personal protection.

## References

[1] Papageorgiou S.N., Koletsi D., Iliadi A., Peltomaki T., Eliades T. (2020). Treatment outcome with orthodontic aligners and fixed appliances: A systematic review with meta-analyses. Eur. J. Orthod..

[2] Pithon M.M., Baião F.C., Sant’ Anna L.I., Paranhos L.R., Cople Maia L. (2019). Assessment of the effectiveness of invisible aligners compared with conventional appliance in aesthetic and functional orthodontic treatment: A systematic review. J. Investig. Clin. Dent..

[3] Ke Y., Zhu Y., Zhu M. (2019). A comparison of treatment effectiveness between clear aligner and fixed appliance therapies. BMC Oral. Health..

[4] Lu H., Tang H., Zhou T., Kang N. (2018). Assessment of the periodontal health status in patients undergoing orthodontic treatment with fixed appliances and Invisalign system: A meta-analysis. Med. Baltim..

[5] Kumar S., Kumar S., Hassan N., Anjan R., Shaikh S., Bhowmick D. (2021). A Comparative Assessment of the Effect of Professional Oral Hygiene Measures on the Periodontal Health of Patients Undergoing Fixed Orthodontic Appliance Therapy. J. Pharm. Bioallied. Sci..

[6] Cerroni S., Pasquantonio G., Condò R., Cerroni L. (2018). Orthodontic Fixed Appliance and Periodontal Status: An Updated Systematic Review. Open. Dent. J..

[7] Yáñez-Vico R.M., Iglesias-Linares A., Ballesta-Mudarra S., Ortiz-Ariza E., Solano-Reina E., Perea E.J. (2015). Short-term effect of removal of fixed orthodontic appliances on gingival health and subgingival microbiota: A prospective cohort study. Acta. Odontol. Scand..

[8] Eltayeb M.K., Ibrahim Y.E., El Karim I.A., Sanhouri N.M. (2017). Distribution of white spot lesions among orthodontic patients attending teaching institutes in Khartoum. BMC Oral. Health..

[9] Pinto A.S., Alves L.S., Maltz M., Zenkner J.E.D.A. (2020). Association between fixed orthodontic treatment and dental caries: A 1-year longitudinal study. Braz. Oral. Res..

[10] Pinto A.S., Alves L.S., Zenkner J.E.D.A., Zanatta F.B., Maltz M. (2017). Gingival enlargement in orthodontic patients: Effect of treatment duration. Am. J. Orthod. Dentofacial. Orthop..

[11] Almansob Y.A., Alhammadi M.S., Luo X.J., Alhajj M.N., Zhou L., Almansoub H.A., Mao J. (2021). Comprehensive evaluation of factors that induce gingival enlargement during orthodontic treatment: A cross-sectional comparative study. Niger. J. Clin. Pract..

[12] Toti Ç., Meto A., Kaçani G., Droboniku E., Hysi D., Tepedino M., Zaja E., Fiorillo L., Meto A., Buci D. (2022). White Spots Prevalence and Tooth Brush Habits during Orthodontic Treatment. Healthcare.

[13] Scheerman J.F.M., Empelen P., Loveren C., Pakpour A.H., Meijel B., Mierzaie Z., Braak M.C.T., Verrips G.H.W., Gholami M. (2017). An application of the Health Action Process Approach model to oral hygiene behaviour and dental plaque in adolescents with fixed orthodontic appliances. Int. J. Paediatr. Dent..

[14] Kim S.H., Choi D.S., Jang I., Cha B.K., Jost-Brinkmann P.G., Song J.S. (2012). Microbiologic changes in subgingival plaque before and during the early period of orthodontic treatment. Angle. Orthod..

[15] Ireland A.J., Soro V., Sprague S.V., Harradine N.W., Day C., Al-Anezi S., Jenkinson H.F., Sherriff M., Dymock D., Sandy J.R. (2014). The effects of different orthodontic appliances upon microbial communities. Orthod. Craniofac. Res..

[16] Guo R., Lin Y., Zheng Y., Li W. (2017). The microbial changes in subgingival plaques of orthodontic patients: A systematic review and meta-analysis of clinical trials. BMC Oral. Health..

[17] Mártha K., Lőrinczi L., Bică C., Gyergyay R., Petcu B., Lazăr L. (2016). Assessment of periodontopathogens in subgingival biofilm of banded and bonded molars in early phase of fixed orthodontic treatment. Acta. Microbiol. Et. Immunol. Hung..

[18] Sioustis I.-A., Martu M.-A., Aminov L., Pavel M., Cianga P., Kappenberg-Nitescu D.C., Luchian I., Solomon S.M., Martu S. (2021). Salivary Metalloproteinase-8 and Metalloproteinase-9 Evaluation in Patients Undergoing Fixed Orthodontic Treatment before and after Periodontal Therapy. Int. J. Environ. Res. Public. Health..

[19] Lazăr L., Bică C.I., Martha K., Păcurar M., Bud E., Lazăr A.P., Lorinczi L. (2017). The use of Polymerase Chain Reaction (PCR) for Indentifying periodontopathogenic bacteria—Therapeutic Implications in periodontal disease. Rev. Chim.Buchar..

[20] Qadri T., Miranda L., Tunér J., Gustafsson A. (2005). The short-term effects of low-level lasers as adjunct therapy in the treatment of periodontal inflammation. J. Clin. Periodontol..

[21] Ezber A., Taşdemir İ., Yılmaz H.E., Narin F., Sağlam M. (2022). Different application procedures of Nd:YAG laser as an adjunct to scaling and root planning in smokers with stage III grade C periodontitis: A single-blind, randomized controlled trial. Ir. J. Med. Sci..

[22] Abduljabbar T., Vohra F., Kellesarian S.V., Javed F. (2017). Efficacy of scaling and root planning with and without adjunct Nd:YAG laser therapy on clinical periodontal parameters and gingival crevicular fluid interleukin 1-beta and tumor necrosis factor-alpha levels among patients with periodontal disease: A prospective randomized split-mouth clinical study. J. Photochem. Photobiol. B..

[23] Akram Z., Abduljabbar T., Sauro S., Daood U. (2016). Effect of photodynamic therapy and laser alone as adjunct to scaling and root planing on gingival crevicular fluid inflammatory proteins in periodontal disease: A systematic review. Photodiagnosis Photodyn. Ther..

[24] Lafzi A., Mojahedi S.M., Mirakhori M., Torshabi M., Kadkhodazadeh M., Amid R., Karamshahi M., Arbabi M., Torabi H. (2019). Effect of one and two sessions of antimicrobial photodynamic therapy on clinical and microbial outcomes of non-surgical management of chronic periodontitis: A clinical study. J. Adv. Periodontol. Implant. Dent..

[25] Theodoro L.H., Marcantonio R.A.C., Wainwright M., Garcia V.G. (2021). LASER in periodontal treatment: Is it an effective treatment or science fiction?. Braz. Oral. Res..

[26] Smiley C.J., Tracy S.L., Abt E., Michalowicz B.S., John M.T., Gunsolley J., Cobb C.M., Rossmann J., Harrel S.K., Forrest J.L. (2015). Systematic review and meta-analysis on the nonsurgical treatment of chronic periodontitis by means of scaling and root planning with or without adjuncts. J. Am. Dent. Assoc..

[27] Chambrone L., Wang H.M., Romanos G.E. (2018). Antimicrobial photodynamic therapy for the treatment of periodontitis and peri-implantitis: An American Academy of Peridontology best evidence review. J. Periodont.

[28] Pawelczyk-Madalinska M., Benedicenti S., Salagean T., Bordea I.R., Hanna R. (2021). Impact of adjunctive diode laser application to non-suurgicel periodontal therapy on clinica, microbiological and immunological outcomes in management of chronic periodontitis: A systematic review of human randomized controlled trials. J. Inflamm. Res..

[29] Chambrone L., Ramos U.D., Reynolds M.A. (2018). Infrared lasers for the treatment of moderate to severe periodontitis: An American Academy of Periodontology best evidence review. J. Periodontol..

[30] Salvi G.E., Stähli A., Schmidt J.C., Ramseier C.A., Sculean A., Walter C. (2020). Adjunctive laser or antimicrobial photodynamic therapy to non-surgical mechanical instrumentation in patients with untreated periodontitis: A systematic review and meta-analysis. J. Clin. Periodontol..

[31] Sanz M., Herrera D., Kebschull M., Chapple I., Jepsen S., Beglundh T., Sculean A., Tonetti M.S., EFP Workshop Participants and Methodological Consultants (2020). Treatment of stage I-III periodontitis: The EFP S3 level clinical practice guideline. J. Clin. Periodontol..

[32] Francis N.C., Yao W., Grundfest W.S., Taylor Z.D. (2017). Laser-Generated Shockwaves as a Treatment to Reduce Bacterial Load and Disrupt Biofilm. IEEE Trans Biomed. Eng..

[33] Rupel K., Zupin L., Ottaviani G., Bertani I., Martinelli V., Porrelli D., Vodret S., Vuerich R., Da Silva D.P., Bussani R. (2019). Blue laser light inhibits biofilm formation in vitro and in vivo by inducing oxidative stress. NPJ Biofilms. Microbiomes..

[34] Gojkov-Vukelic M., Hadzic S., Dedic A., Konjhodzic R., Beslagic E. (2013). Application of a diode laser in the reduction of targeted periodontal pathogens. Acta Inf. Med..

[35] Grzech-Leśniak K., Matys J., Dominiak M. (2018). Comparison of the clinical and microbiological effects of antibiotic therapy in periodontal pockets following laser treatment: An in vivo study. Adv. Clin. Exp. Med..

[36] Wang Y., Li W., Shi L., Zhang F., Zheng S. (2017). Comparison of clinical parameters, microbiological effects and calprotectin counts in gingival crevicular fluid between Er: YAG laser and conventional periodontal therapies: A split-mouth, single-blinded, randomized controlled trial. Med. Baltim..

[37] Lopes B.M., Theodoro L.H., Melo R.F., Thompson G.M., Marcantonio R.A. (2010). Clinical and microbiologic follow-up evaluations after non-surgical periodontal treatment with erbium:YAG laser and scaling and root planing. J. Periodontol..

[38] Milne T., Coates D., Leichter J., Soo L., Williams S., Seymour G., Cullinan M. (2016). Periodontopathogen levels following the use of an Er:YAG laser in the treatment of chronic periodontitis. Aust. Dent. J..

[39] Calderín S., García-Núñez J.A., Gómez C. (2013). Short-term clinical and osteoimmunological effects of scaling and root planing complemented by simple or repeated laser phototherapy in chronic periodontitis. Lasers. Med. Sci..

[40] Dalvi S., Benedicenti S., Sălăgean T., Bordea I.R., Hanna R. (2021). Effectiveness of Antimicrobial Photodynamic Therapy in the Treatment of Periodontitis: A Systematic Review and Meta-Analysis of In Vivo Human Randomized Controlled Clinical Trials. Pharmaceutics.

[41] Sufaru I.-G., Martu M.-A., Luchian I., Stoleriu S., Diaconu-Popa D., Martu C., Teslaru S., Pasarin L., Solomon S.M. (2022). The Effects of 810 nm Diode Laser and Indocyanine Green on Periodontal Parameters and HbA1c in Patients with Periodontitis and Type II Diabetes Mellitus: A Randomized Controlled Study. Diagnostics..

[42] Ren C., McGrath C., Gu M., Zhang C., Kumoi F.H. (2020). Low-level laser therapy-aided orthodontic treatment of periodontally compromised patients: A randomized controlled trial. Lasers. Med. Sci..

[43] Abellan R., Gomez C., Oteo M.D., Scuzzo G., Palma J.C. (2016). Short- and medium-term effects of low-level laser therapy on periodontal status in lingual orthodontic patients. Photomed. Laser Surg..

